# Tailoring polyvinyl alcohol-sodium alginate (PVA-SA) hydrogel beads by controlling crosslinking pH and time

**DOI:** 10.1038/s41598-022-25111-7

**Published:** 2022-12-02

**Authors:** Pieter Candry, Bruce J. Godfrey, Ziwei Wang, Fabrizio Sabba, Evan Dieppa, Julia Fudge, Oluwaseyi Balogun, George Wells, Mari-Karoliina Henriikka Winkler

**Affiliations:** 1 Civil and Environmental Engineering, University of Washington, 201 More Hall, Box 352700, Seattle, WA 98195-2700 USA; 2 Mechanical Engineering Department, Northwestern University, Evanston, IL 60208 USA; 3 Black & Veatch, New York, NY USA; 4 Theoretical and Applied Mechanics Program, Northwestern University, Evanston, IL 60208 USA; 5 Civil and Environmental Engineering Department, Northwestern University, Evanston, IL 60208 USA

**Keywords:** Chemical engineering, Gels and hydrogels, Biotechnology

## Abstract

Hydrogel-encapsulated catalysts are an attractive tool for low-cost intensification of (bio)-processes. Polyvinyl alcohol-sodium alginate hydrogels crosslinked with boric acid and post-cured with sulfate (PVA-SA-BS) have been applied in bioproduction and water treatment processes, but the low pH required for crosslinking may negatively affect biocatalyst functionality. Here, we investigate how crosslinking pH (3, 4, and 5) and time (1, 2, and 8 h) affect the physicochemical, elastic, and process properties of PVA-SA-BS beads. Overall, bead properties were most affected by crosslinking pH. Beads produced at pH 3 and 4 were smaller and contained larger internal cavities, while optical coherence tomography suggested polymer cross-linking density was higher. Optical coherence elastography revealed PVA-SA-BS beads produced at pH 3 and 4 were stiffer than pH 5 beads. Dextran Blue release showed that pH 3-produced beads enabled higher diffusion rates and were more porous. Last, over a 28-day incubation, pH 3 and 4 beads lost more microspheres (as cell proxies) than beads produced at pH 5, while the latter released more polymer material. Overall, this study provides a path forward to tailor PVA-SA-BS hydrogel bead properties towards a broad range of applications, such as chemical, enzymatic, and microbially catalyzed (bio)-processes.

## Introduction

Hydrogel beads are a powerful tool for (bio)-process engineering. These hydrated polymer matrices can be engineered to encapsulate chemical, enzymatic, and live microbial catalysts to drive desired chemical conversions^1–3^. While hydrogels can be employed in a similar fashion to granular biofilms (or, granular sludge), they uniquely offer the possibility to combine organisms with complementary metabolic traits to achieve desired functionalities^4,5^. Additionally, tailoring hydrogel bead size can optimize the effective catalytic volume of bioprocesses and select for specific populations of interest^6,7^. Together, the potential to minimize process footprint and capital investment^8^ makes hydrogels an attractive venue for bio-process intensification^9,10^.

Polymer composition and cross-linking chemistry are critical in the development, design, and application of hydrogels. The selected polymer and its final concentration will govern the physicochemical properties of hydrogels, such as diffusivity and viscoelastic characteristics^11–14^. These properties are critical to the eventual efficiency and durability of a hydrogel-based process. Moreover, when microbial catalysts are embedded in hydrogels, the cytotoxicity of hydrogel monomers and the cross-linking chemistry should be considered to minimize cell loss^15,16^. Similarly, the micron-scale structure of the hydrogel matrix may confine microbes, resulting in crowded hydrogels that alter the phenotypic properties of the embedded cells, and eventually rupturing the hydrogel^17^. Consequently, establishing approaches to tailor the physicochemical properties of hydrogel beads made up of known non-toxic matrices may significantly improve their utility in bioprocess engineering.

Two widely applied hydrogel polymers for encapsulating microbial catalysts are sodium alginate (SA) and polyvinyl alcohol (PVA). Alginate is a natural carbohydrate polymer that can be crosslinked with Ca^2+^-ions (e.g., in a CaCl_2_ bath), providing an encapsulation process with low stress for microbial catalysts^18,19^. Unfortunately, these hydrogels are mechanically weak and dissolve when exposed to high concentrations of monovalent cations or Ca-chelators such as phosphate, citrate, or EDTA^20^. Combining crosslinking of SA by Ca^2+^ with boric acid catalyzed PVA-crosslinking at low pH has been shown to produce more robust hydrogel beads^21^. PVA is a low-cost polymer that is non-toxic to microbial cells^22^, enabling the successful application of these hybrid PVA-SA hydrogels in bioprocess and environmental engineering applications^10^.

To improve the applicability of these PVA-SA hydrogels, different tailoring approaches have been developed. First, the mechanical and chemical stability of PVA-SA beads can be increased by utilizing higher concentrations of PVA and boric acid during crosslinking, although this does come at the expense of the diffusivity of the matrix^23,24^. Second, post-curing the produced beads with anions that can crosslink or enhance hydrogen-bonding interactions between PVA chains (e.g., phosphate, sulfate) may further improve hydrogel properties^25,26^. Post-curing with sulfate was found to be particularly effective to produce stronger, more stable beads^25,26^. These PVA-SA-boric acid-SO_4_^2−^ (PVA-SA-BS) hydrogels reportedly contain 10–100 µm-scale pores^27,28^, and have very high diffusion rates for small molecules^29,30^. Together, the physicochemical properties of these chemically tailored PVA-SA-BS provide potential for bioprocess applications.

A critical issue with current procedures for PVA-SA-BS hydrogel production is that the low pH (~ 3) of saturated boric is harmful to cells, often requiring weeks-long recovery times after hydrogel production^29,31^. Production of PVA-SA-BS hydrogels at pH closer to neutral could increase survival rate of biocatalysts and improve process start-up times but it is unclear what the impact of higher pH is on the acid-catalyzed crosslinking mechanism. Similarly, reducing the exposure time to boric acid may also reduce cell stress^27^, but may impact critical properties of the produced beads. To date, no study has comprehensively investigated how crosslinking pH and duration affect the properties of the resulting PVA-SA-BS hydrogel beads. Moreover, multifaceted analyses of physical, mechanical, chemical, and process properties of PVA-SA-BS hydrogel beads are also absent in literature despite their interdependence and the need to understand how changing production parameters may induce trade-offs in desirable properties. Here, we investigate if crosslinking pH (3, 4, and 5) and time (1, 2, and 8 h) alter the properties of PVA-SA-BS hydrogel beads, with the intent of developing simple procedures to predictably tailor these versatile, biocompatible hydrogels.

## Results and discussion

### Physical properties of PVA-SA-BS hydrogels produced under different conditions

**Figure 1:**
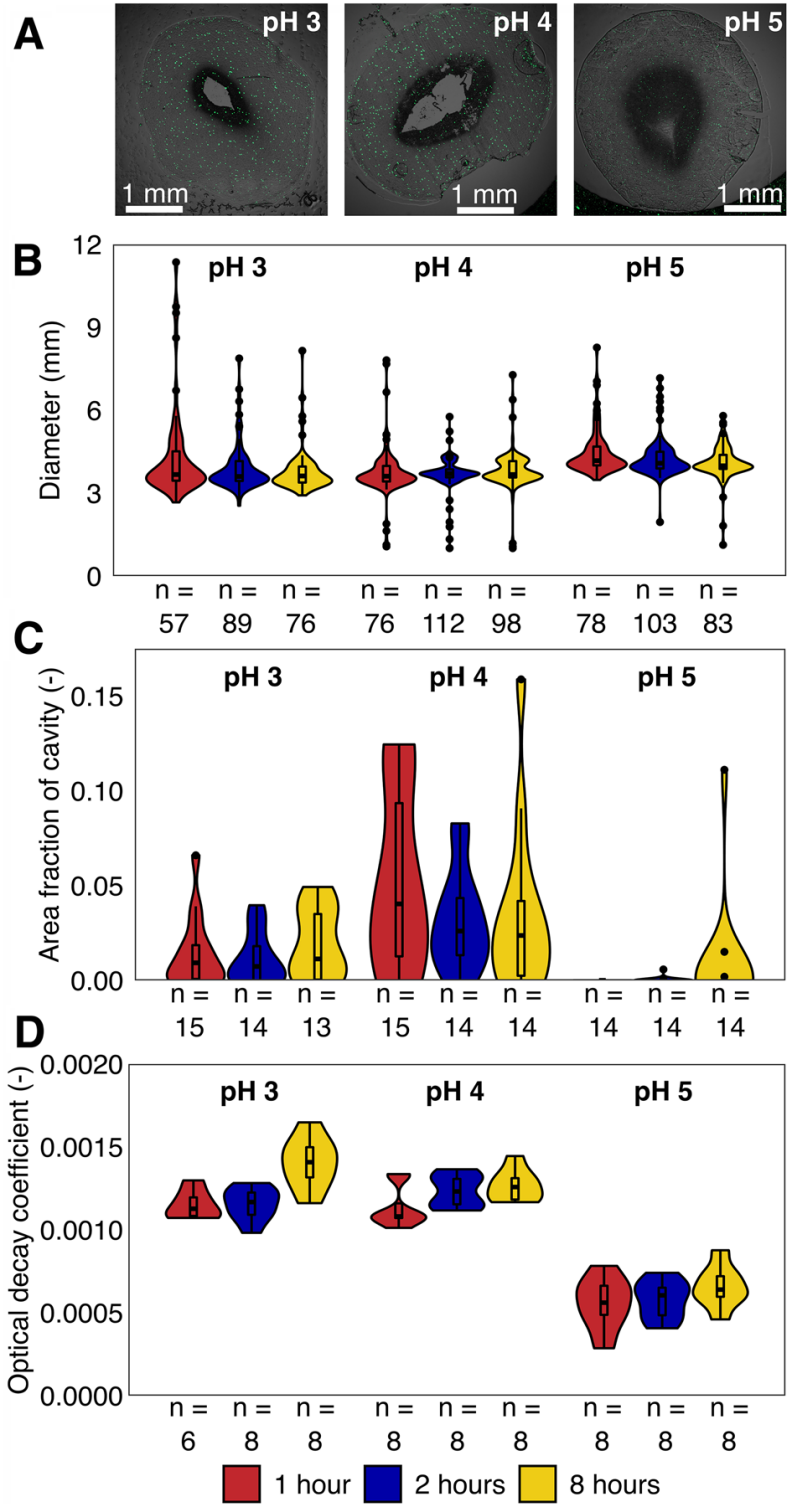
Physical properties of hydrogels produced at 3 different pH (pH 3, 4, and 5) and 3 different crosslinking times (1, 2, and 8 h). (**A**) shows composite light and green fluorescence microscopic images of representative hydrogel sections for beads produced at pH 3, 4, and 5. (**B**–**D**) represent respectively bead diameter distribution, internal cavity area from section images, and the optical decay coefficient derived from optical coherence tomography measurements. Violin plots are normalized to equal widths across all conditions.

The impact of different bead production procedures (i.e., crosslinking pH and time) on the physical properties of beads was evaluated, focusing on bead sizes, internal cavities in beads (Fig. 1A) and polymer cross-linking density in beads.

Bead sizes ranged from 1 mm to over 10 mm (Fig. 1B), with median bead sizes ranging from 3.6 to 4.18 mm over different production conditions. Non-parametric anova showed that crosslinking pH was the strongest determinant of bead size ranks (R^2^ = 0.062, p = 0.001). Crosslinking time also had a significant, but smaller impact (R^2^ = 0.014, p = 0.004). Pairwise Wilcox rank-tests confirmed that beads produced at pH 5 (d_median_ = 3.99–4.17 mm) had significantly larger median bead sizes than all beads produced at pH 4 (d_median_ = 3.61–3.71 mm, p < 0.001) and beads polymerized for 2 or 8 h at pH 3 (d_median_ = 3.60–3.62 mm, p < 0.001).

Microscopic visualization of hydrogel bead cryosections revealed that some beads contained internal cavities (Fig. 1A). The area fraction of these cavities within a section showed large variability across production conditions, ranging from no observed cavities to 15% of the total section area (Fig. 1C). Beads produced at pH 4 had the largest median cavity area fractions (1 h, 4.0%; 2 h, 2.6%, 8 h, 2.4%), followed by pH 3 (1 h, 0.9%; 2 h, 0.7%; 8 h, 1.1%) and pH 5 (1, 2, and 8 h, 0.0%). The only significant differences in median cavity areas were observed between beads produced at pH 5 and those produced at pH 3 or 4, although these differences were not uniform across all pairwise comparisons (Table S.2).

Optical decay coefficients were derived from OCT-measurements and reflect bead opacity and cross-linking density in hydrogel beads. Both crosslinking time and pH affected optical decay coefficients (p < 0.001) with pH having the largest impact (R^2^ = 0.82) while time had a smaller impact (R^2^ = 0.04). Beads produced at pH 5 had significantly lower decay coefficients than beads produced at pH 3 and 4 (p < 0.001 across all pairwise comparisons, Table S.3), implying pH 5 beads were more translucent and less densely cross-linked. Decay coefficients in beads produced at pH 3 and 4 were more similar, with only beads polymerized for 8 h at pH 3 being higher than other pH 3 beads, as well as higher than beads polymerized for 1 h at pH 4 (p < 0.01, Table S.3).

Crosslinking pH significantly affected PVA-SA-BS hydrogel beads’ physical properties, suggesting that pH may alter PVA-SA crosslinking chemistry. Moreover, the small impact of time indicates that the initial stages of crosslinking govern the eventual morphology. During hydrogel production, a PVA-SA droplet is dropped into the crosslinking bath (5% boric acid and 0.7% Ca^2+^). These crosslinkers will first act on the periphery of the droplet, creating a thin hydrogel shell. This shell can then limit the diffusion of crosslinker^13^, slowing down subsequent crosslinking. In beads with higher crosslinking densities (i.e., pH 3 and 4 beads, Fig. 1D), such diffusion limitations may have strongly impeded further crosslinking, resulting in the observed internal cavities (Fig. 1A,C). Consequently, the physical properties of the hydrogel beads are dependent on the rates and chemistry of crosslinking. The dependency of physical properties on crosslinking chemistry is further exemplified by pairing opacity and bead size data (Fig. 1B,D). Beads at pH 5 had significantly lower optical decay coefficients and displayed fewer cavities, suggesting an altered polymer chemistry. This altered polymer chemistry may have been due to reduced Ca-crosslinking of alginate at low pH. Ca-crosslinking of alginate relies on the anionic species, while estimates of the pKa for alginic acid range from 2.84 to 4.36^32,33^. Higher pH may therefore increase alginate crosslinking, altering the hydrogel polymer make-up, as well as the potential for more complete crosslinking throughout the whole hydrogel. Changes in polymer make-up and polymerization conditions (e.g., boric acid concentration, post-curing, etc.) have been shown to affect swelling behavior of hydrogels^26,27^. We therefore hypothesize crosslinking at different pH altered polymer make-up, modulating crosslinking densities (i.e., opacity, Fig. 1D) as well as swelling behavior (i.e., observed size, Fig. 1B).

These data show crosslinking pH is a key driver of the physical properties of PVA-SA-BS hydrogel beads, which may be associated with shifts in crosslinking processes during the initial stages of bead formation.

### Elastic properties of PVA-SA-BS hydrogels produced under different conditions

**Figure 2:**
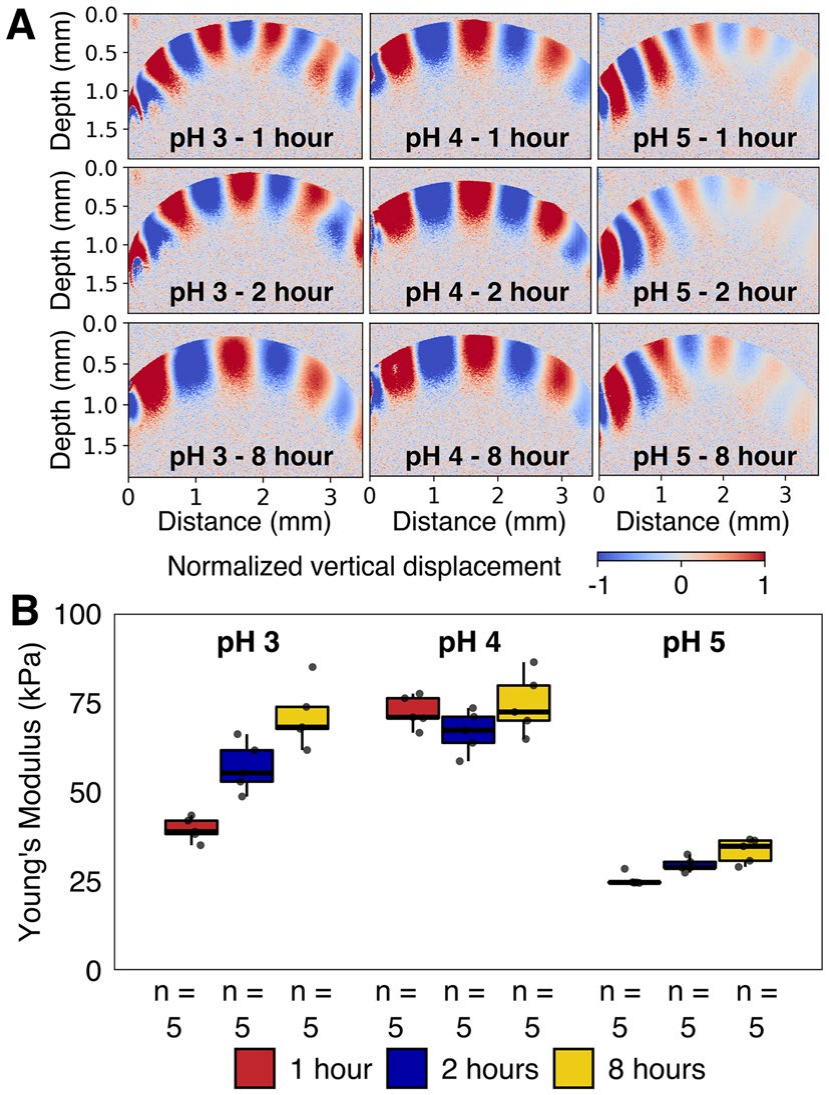
Elasticity of hydrogel beads. (**A**) shows representative optical coherence elastography (OCE) images obtained at 4.0 kHz for each of the conditions. The piezoelectric transducer was located at the left of the image. (**B**) shows the Young’s modulus measured at different frequencies (2–6 kHz, grey dots) and the data distributions (box plots).

Young’s modulus (E) was determined with optical coherence elastography (OCE) as a metric of bead elasticity during elastic deformation (Fig. 2). Both crosslinking pH and time significantly affected Young’s moduli (p < 0.001), with pH having the largest impact (R^2^ = 0.77), while crosslinking time affected Young’s moduli less strongly (R^2^ = 0.09). Beads produced at pH 5 were the softest among all conditions, with significantly lower E-values (approx. 60% decrease) than beads produced at pH 4 (Fig. 2B). Moreover, pH 5 beads were also significantly softer than beads polymerized at pH 3 for 2- or 8-h (p < 0.001, Table S.4). Notably, beads produced at pH 3 became significantly stiffer with longer crosslinking times (p < 0.01, Table S.4), but no such significant trend could be observed for beads produced at pH 4 and 5. Compared to other materials, PVA-SA-BS hydrogels had 3 orders of magnitude higher E-values than natural biofilms (40–100 Pa^34–36^), and two to sixfold higher than granular biofilms (12 kPa^37^), confirming these hydrogels are robust materials for bioprocess applications (Table 1). Comparing E-values of PVA-SA-BS hydrogels to its constituent gels (i.e., alginate or PVA) did not reveal clear patterns due to the large impact of polymer concentration and crosslinking method. However, both PVA-only and SA-only hydrogels can be much stiffer than the PVA-SA-BS hydrogels produced here^13,38^. This highlights that the interpenetrating network of PVA and SA polymers obtained by PVA-SA-BS hydrogel production behaves differently than the individually crosslinked polymers^39^. Last, Young’s moduli of PVA-SA-BS hydrogels were an order of magnitude higher than literature reports for EPS-derived hydrogels, were similar to carrageenan-gels, and were an order of magnitude lower than solidified agar (Table 1). The sulfate treatment applied uniformly across all beads here was previously shown to significantly improve process durability of PVA-SA-BS hydrogels produced at pH 3^29^. Overall, PVA-SA-BS hydrogels produced at pH between 3 and 5 are robust materials with properties similar to many existing natural and synthetic hydrogel-like materials. Moreover, controlling crosslinking pH and time can enable tailoring the viscoelastic properties of PVA-SA-BS beads towards different bioprocess applications.

**Table 1:** Young’s moduli (E) of natural and synthetic hydrogel-like materials.

Substrate		Measurement method	E (kPa)	Ref.
Biofilms	Natural biofilms	Shear deformation and OCT	0.036	^34^
		Shear deformation and microscopy	0.04	^35^
		Shear deformation and microscopy	0–0.1	^36^
	Granular biofilms	Parallel disc rheometer	12	^37^
Hydrogels	Alginate 2%	Uniaxial compression	460	^13^
	Alginate 2–5%	Uniaxial compression	250–600	^14^
	Alginate 2.5%	Parallel disc rheometer	30	^40^
	Alginate 10%	Uniaxial compression	2600–3400	^13^
	PVA—freeze–thaw	Tensile test	10	^41^
	PVA—DMSO-boric acid	Tensile test	4820–5140	^38^
	EPS	Rheometer	1–5	^40^
	Carrageenan 1.5–2.5%	Uniaxial compression	63–90	^42^
	Agar 1.5–2.5%	Uniaxial compression	140–260	^42^
	PVA-SA-BS	OCE	25.4–74.7	Figure 2

### Diffusive properties of PVA-SA-BS hydrogels produced under different conditions

**Figure 3:**
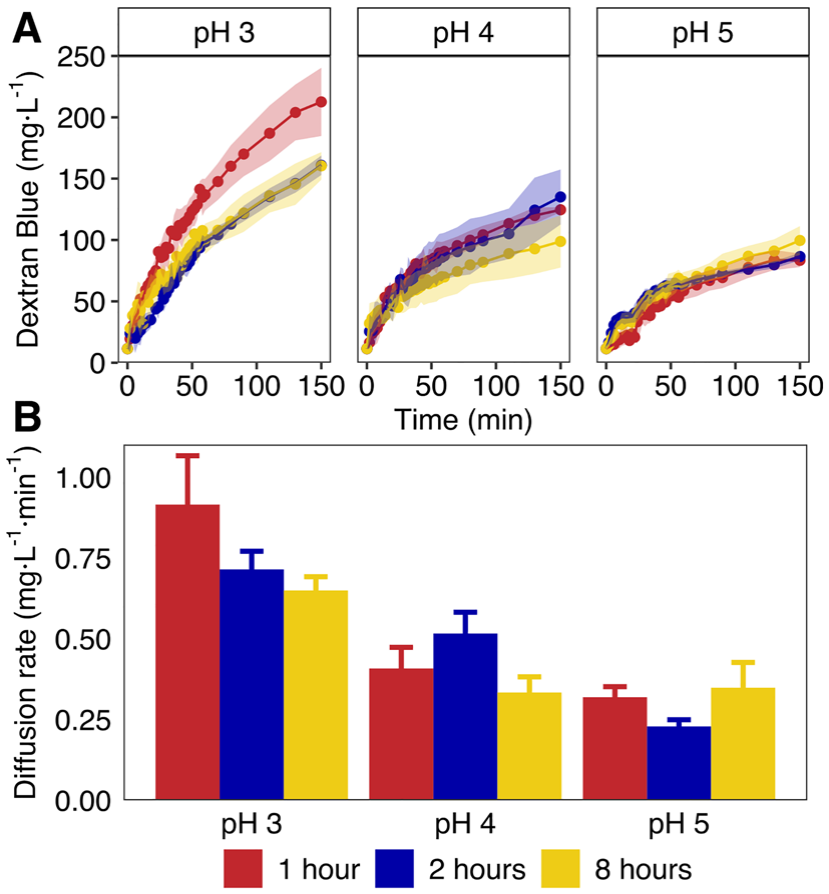
Diffusion of Dextran Blue out of hydrogel beads produced under varying pH and crosslinking times. Dextran Blue concentration was measured as OD600 in glass tubes. (**A**) represents Dextran Blue concentration profiles over time for each type of bead; (**B**) represents diffusion rate of Dextran Blue from each bead type, determined as the slope of the concentration profile over the last 100 min.

Diffusion of Dextran Blue out of hydrogels was used as a proxy for diffusion of macromolecular complexes (e.g., enzymes) in hydrogels (Fig. 3). Specifically, we can interpret the total amount of Dextran Blue released by a hydrogel after saturation as a proxy for pore space^43^. Similarly, rates of Dextran Blue diffusion are the result of both polymer make-up (i.e., diffusion rates through the polymer itself) as well as pore architecture-factors such as pore size^43^, and pore connectivity.

Total Dextran Blue released at the end of the assay decreased with increasing crosslinking pH, indicating that beads produced at lower pH may have larger internal pore spaces (Fig. 3A). Beads produced at pH 3 consistently released significantly more Dextran Blue than those produced at pH 5, as well as beads produced at pH 4 with 8 h crosslinking time (p < 0.01, Table S.5). Moreover, beads polymerized for 1 h at pH 3 released more Dextran Blue than any other bead type (p < 0.05), indicating these beads may have had pore architectures that favor higher diffusion rates of macro-molecular structures.

Rates of Dextran Blue diffusion over the last 100 min of the diffusion assay showed similar trends across crosslinking pH and times (Fig. 3B). Beads at pH 3 had the highest diffusion rates of Dextran Blue, with consistently significantly higher values than beads at pH 5, and beads polymerized at pH 4 for 1 and 8 h (p < 0.05, Table S.6). Again, beads produced at pH 3 for 1 h had the highest rate of Dextran Blue diffusion, with significantly higher values than all other beads, except those polymerized for 2 h at pH 3 (p < 0.01).

Hydrogel polymer networks impose diffusion limitations for chemicals diffusing from the bulk aqueous phase in, or from the hydrogel outwards. In engineering applications, the balance between diffusion rates and reaction rates is critical to maximize the effective catalytic volume of the system (i.e., Thiele modulus). Moreover, in applications leveraging microbes and complex microbial communities, such diffusion limitations enable supporting desired functionalities in the hydrogels^4^. While these applications benefit from establishing absolute diffusion coefficients (D), determination of D is limited by (i) the spherical nature of the hydrogels, and (ii) the heterogeneous inner structure of the hydrogels (i.e., cavities). Solutions have been proposed for the former issue^23^, however, the latter issue would require better characterization of the pore architecture (i.e., pore space, size, and connectivity), as well as the diffusive properties of the liquid contained in the internal cavities. On the other hand, determining D in flat sheets of PVA-SA-BS hydrogels^29^ would overlook the impact of droplet-based crosslinking on hydrogel morphology. Ultimately, crosslinking pH strongly alters the diffusive properties of PVA-SA-BS hydrogels, a critical design factor for (bio)processes.

### Process performance of PVA-SA-BS hydrogels produced under different conditions

**Figure 4:**
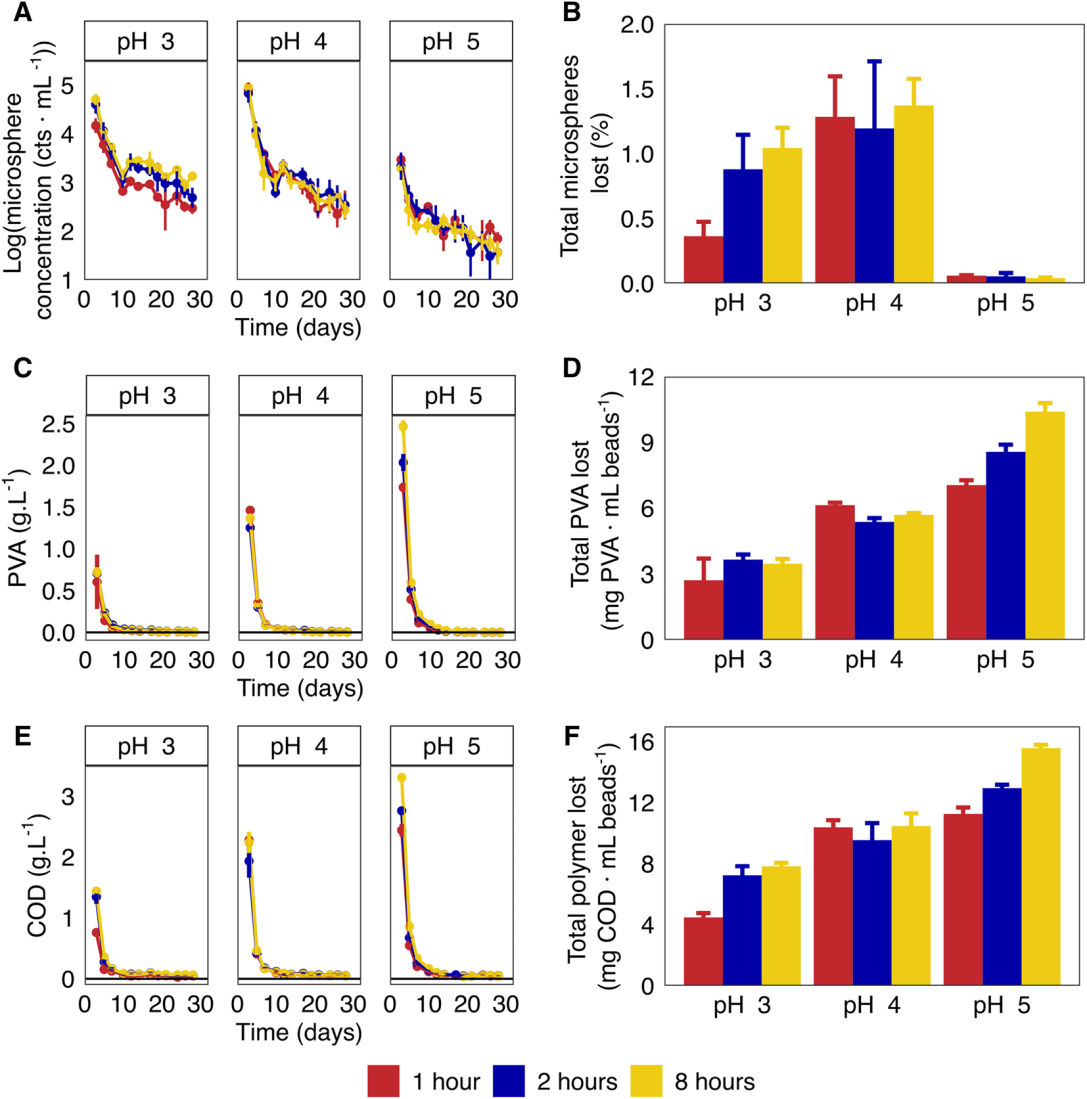
Loss of microbial cell proxies and hydrogel building blocks during a 30-day incubation. (**A**) and (**B**) show daily and total loss of fluorescent microspheres from hydrogel beads respectively. (**C**) and (**D**) show daily and total loss of PVA from hydrogels, and (**E**) and (**F**) show daily and total loss of polymer (expressed as chemical oxygen demand (COD)) from hydrogels.

In a simulated hydrogel bead process, the loss of fluorescent microspheres (d = 1 µm) as a proxy for cell retention as well as the loss of hydrogel building blocks was monitored to investigate how different hydrogel production conditions affect the process behavior of beads (Fig. 4).

Microsphere loss was highest in the initial liquid exchange steps (Days 0–7), but rapidly dropped by 2 orders of magnitude over this period (Fig. 4A). After this initial week, microsphere concentrations continued to decrease, albeit at a slower rate. Throughout the experiment, the supernatant of pH 5 beads typically contained an order of magnitude less microspheres than beads produced at pH 3 or pH 4 (Fig. 4A). As a result, the total microsphere loss over the entire incubation (Fig. 4B) from beads produced at pH 5 was significantly lower than from beads produced at pH 4 (p < 0.001; Table S.7), and beads polymerized at pH 3 for 2 and 8 h (p < 0.05 and p < 0.01 respectively). Larger, and more connected micropores will facilitate faster exchange between the intra-hydrogel and bulk aqueous phase for both Dextran Blue (2000 kDa) and fluorescent microspheres acting as cell proxies (1 μm). This interpretation provides context for the observation that hydrogels produced at pH 5 lost the least cells and had the lowest diffusion rates, indicating the matrix may be more uniform and gel-like, resembling alginate hydrogels^44^. On the other hand, PVA-SA-BS hydrogels crosslinked at pH 3 have been observed to be more sponge-like with 10–100 µm-scale pores^27^, explaining their higher rates of Dextran Blue diffusion and microsphere loss. It should be noted that other processes may also have controlled cell loss. Beads polymerized at pH 3 for 1 h had higher diffusivities (Fig. 3) and lower microsphere loss compared to beads produced at pH 4 (p < 0.05), suggesting the relation between cell loss and Dextran Blue diffusion rates may not be fully linear. Moreover, fluorescent microspheres acted as proxies for cells, but can only mimic passive cell loss and not active outgrowth of microbial cells from the hydrogel matrix. This may be exacerbated by potential differences in surface chemistry and particle charge of these microspheres compared to microbial cells, which might also affect abiotic interactions with the hydrogel matrix. Regardless, the observed trends suggest that crosslinking pH controls the micrometer-scale pore architecture of PVA-SA-BS hydrogel matrices, altering their capacity to retain microbial cells.

Hydrogel building blocks were also released from the hydrogels during the initial phase. Similar to cell loss, the initial 7 days of incubation released the highest concentrations of hydrogel monomers, with hydrogels produced at higher pH releasing more polymer (Fig. 4C,E). Initial concentrations of PVA in the effluent ranged from 604 ± 324 mg PVA L^−1^ (pH 3–1 h) to 2.46 ± 0.07 g PVA L^−1^ (pH 5–8 h), while total hydrogel monomer (measured as COD) ranged from 760 ± 7 mg COD L^−1^ (pH 3–1 h) to 3.31 ± 0.02 g COD L^−1^ (pH 5–8 h). Across all conditions, the initial effluent contained much higher COD-loads than most domestic wastewaters^45^, implying that effluent from start-up phases of (full-scale) hydrogel processes should be treated separately. Trends for both PVA and total hydrogel monomer loss over the entire incubation are similar (Fig. 4D,F), with PVA-loss increasing significantly with increasing crosslinking pH (p < 0.01; Table S.8), while trends in total polymer loss (as COD) were less consistent across crosslinking pH (Table S.9). Previous research observed PVA-SA-BS hydrogel beads produced at pH 3 lost 0.026–0.073% of their mass over a 5-week shaking incubation without liquid exchange^29^, similar to the total PVA-losses observed here (0.03–0.09%, Fig. 1D).

Varying crosslinking pH created a clear trade-off in the process properties, where beads that better retained cells, lost more polymer across the initial phases of the process. Mapping such trade-offs is critical to tailor PVA-SA-BS hydrogel beads towards different process applications.

### Tailoring PVA-SA-BS hydrogels for (bio)-process applications

**Figure 5:**
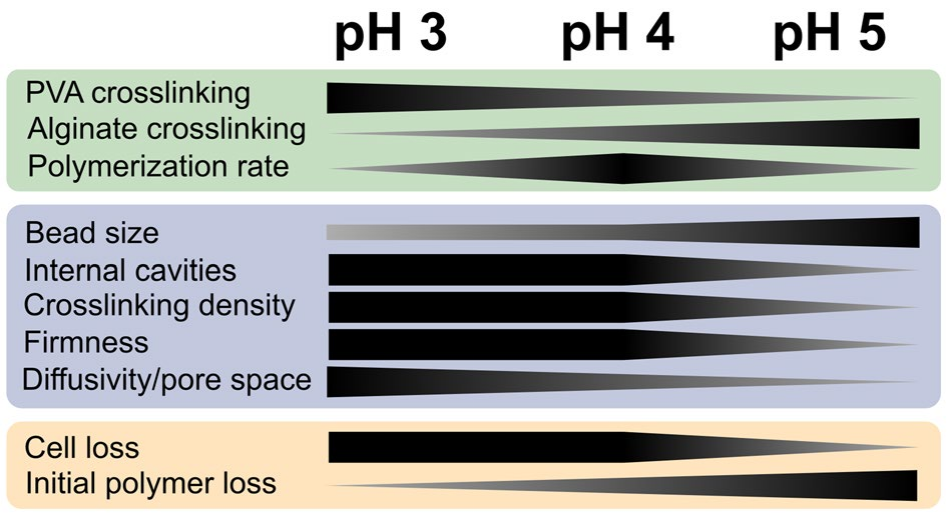
Tailoring physical and functional properties of PVA-SA-BS hydrogels by tuning crosslinking pH.

PVA-SA-(BS) hydrogels are attractive for bioprocess applications incorporating live cells and pure enzymes due to the low toxicity of the crosslinking process^4,46^, and the formation of a matrix enabling high diffusion rates and microbial growth^27,29^. Here, we present the first study quantifying the impact of crosslinking pH and time on a range of physicochemical, elastic, and process properties of PVA-SA-BS hydrogel beads. This unique approach not only enables identifying ideal hydrogel production conditions, but, also identifies trade-offs in desirable hydrogel properties which may be the result of changes in crosslinking and hydrogel chemistry under different production conditions. Overall, pH was the largest determinant of bead morphology, rheology, and process behavior, which could be associated with changes in the crosslinking chemistry of PVA and/or SA. While these chemical mechanisms cannot be confirmed based on the data presented here, there are clear consequences of these changes on the application of these hydrogels in (environmental) engineering settings (Fig. 5).

Crosslinking pH induces trade-offs between microsphere loss and (bio)-catalyst viability on the one hand (better at pH 5), and diffusion rates and bead firmness on the other (better at pH 3/4). These trade-offs can guide the selection of optimal pH for PVA-SA-BS hydrogel production. In applications employing whole-cell microbial catalysts, pH 5-produced beads may be an attractive option. Cell loss may be significantly lower than for PVA-SA-BS beads produced at lower pH, which will help (i) maximize biomass density in the system, and (ii) minimize downstream filtration needs. At the same time, crosslinking at pH 5 might induce less cell stress—depending on the catalyst considered—, reducing start-up time and improving catalytic reaction rates^29,31^. However, depending on the chemistry of the targeted process, lower diffusion rates of pH 5 beads may limit reaction rates, increasing the necessary process footprint and capital investment, making these beads less desirable in some applications. Furthermore, the softer beads might also degrade faster, requiring more frequent replacement in the process. Last, the impact of internal cavities formed at lower crosslinking pH on microbial catalysts is unclear. It may be that such cavities give microbes space to grow without losing cell retention, although specific experiments probing such questions would be necessary to confirm this.

In applications leveraging micron-scale chemical catalysts, low crosslinking pH may be less of an issue than it is for microbial catalysts. Consequently, selecting crosslinking pH then hinges on the trade-off between catalyst retention, diffusivity, and bead degradation. Depending on the chemical catalyst included, catalyst retention may be more significant for chemical catalysts, since they cannot self-replicate like microbial catalysts. One recent report also demonstrated the combination of both microbial and chemical catalyst in a single hydrogel bead for portable urine treatment^47^, further requiring consideration of trade-offs between catalyst retention and diffusion rates.

Last, these trade-offs may further shift in applications utilizing enzymatic catalysts. Catalytic enzymes may behave more similar to the 2000 kDa-sized Dextran Blue, suggesting that their retention may be improved in pH 5-produced PVA-SA-BS hydrogels, although this would have to be confirmed experimentally. Moreover, retention of enzymes in hydrogels can also be altered chemically with, for instance, glutaraldehyde cross-linking, opening the door to opportunities combining high diffusion and catalyst retention^48^.

Overall, we demonstrate that crosslinking pH is a powerful approach to tailor properties of PVA-SA-BS hydrogels that are critical to optimize (bio)-processes and environmental technologies. The trade-offs between cell retention, diffusion rates, and bead firmness are particularly critical factors to maximize process efficiency and durability.

## Conclusions

PVA-SA-BS hydrogels are promising materials for intensification of (bio)-process engineering in a range of applications. Here, we investigate the impact of crosslinking pH and time on a wide range of relevant properties of these hydrogels, including physical, physicochemical, elastic, and process properties. This approach highlighted that pH was the most critical factor influencing hydrogel properties, which may be associated with crosslinking rates of PVA and SA being inversely influenced by pH. This had significant outcomes across all measured properties. Beads produced at lower pH showed unique internal cavities, higher matrix opacity, and smaller bead sizes, which may be associated with diffusion limitation of crosslinking reactants during hydrogel bead production. Low pH beads were also firmer—as measured with OCE—suggesting that stronger PVA-crosslinking may have contributed to hydrogel robustness. These more gel-like beads produced at high pH (more alginate, lower opacity) also showed (i) smaller internal pore space, (ii) lower diffusion rates of Dextran Blue, reflecting micropore architecture, (iii) improved cell retention, and (iv) higher polymer release during initial process phases. These findings present prime opportunities to tailor bead properties in a simple, but effective manner to broaden the application range of PVA-SA-BS hydrogel beads encapsulating chemical, enzymatic, and/or microbial catalysts in bioprocess engineering.

## Materials and methods

### PVA-SA-BS hydrogel production

Boric acid-crosslinked PVA and calcium-crosslinked alginate hydrogels post-cured with sulfate were produced according to Landreau et al.^29^. Briefly, a solution was made containing 100 g L^−1^ PVA (89–98 kDa, Sigma-Aldrich, USA), 10 g L^−1^ SA (BioReagent grade from brown algae, Sigma-Aldrich, USA) and yellow-green fluorescent carboxylate-modified polystyrene 1 µm microspheres (FluoSpheres™, ThermoFisher Scientific, USA) acting as surrogates for microbial cells at a concentration of 1.41·10^7^ microspheres·mL^−1^. This mixture was pumped to a homemade bead-dropping device, consisting of ports with 24G needles dripping polymer droplets into a crosslinking solution containing 50 g H_3_BO_3_ L^−1^ and 26.5 g CaCl_2_⋅2H_2_O L^−1^ stirred with an overhead stirrer. The ratio of total polymer-to-crosslinking solution was fixed at 1:10 across conditions. To investigate the impact of pH on hydrogel formation, the initial pH of the crosslinking solution was set at 3, 4, or 5. To investigate the impact of crosslinking time on hydrogel formation, beads produced at each pH were harvested 1, 2, and 8 h after dropping. After harvesting, beads were strained on a 600 µm sieve, washed thoroughly with DI water to remove the crosslinking solution, and incubated overnight in a solution of 73 g Na_2_SO_4_ L^−1^. This was followed by straining on a 1 mm sieve, washing with DI water, incubating the beads for 8 h in DI water to allow sulfate to diffuse out, and another straining and washing step. After the second wash step, beads were stored in a medium adapted from previous lab studies for wastewater technologies^49^, modified to exclude carbon and nitrogen to minimize bacterial growth, as well as phosphate to reduce bead disintegration via calcium leaching. Specifically, the medium contained per liter: 43.4 mg MgSO_4_⋅7H_2_O, 35.0 mg KCl, 368.2 mg NaCl and 1 mL trace element solution^50^. Beads were stored in the dark at room temperature until the beginning of the experiment.

### Physical properties of hydrogels

Hydrogel bead size was analyzed by distributing beads in a Petri dish and imaging with a Sony Alpha 7C camera. Particle size distributions of at least 57 granules per production condition were calculated using the Color Threshold & Analyze Particle functions in Fiji^51^.

Internal cavities in the bead were analyzed by cryo-sectioning and microscopic visualization. Strained and briefly dried beads were left to incubate overnight in Neg-50 section medium (Richard-Allan Scientific, Kalamazoo, USA) at 4 °C, after which beads were frozen at − 20 °C. Individual beads were sectioned into 5 µm sections using a CryoStar NX50 cryotome. Epifluorescence microscopy was used to capture both light microscopy images of the bead and green fluorescence images of the encapsulated fluorescent microspheres. Total section areas and internal cavity areas were measured by manually circling areas of interest with digital pen on an iPad Pro tablet in ImageJ.JS, an online version of ImageJ^52^.

### Elastic properties of hydrogel beads

Elastic properties of hydrogel beads were characterized by a dynamic OCE method^53–55^. Elastic waves were generated in the sample with a harmonically modulated piezoelectric transducer, and the local steady-state displacement of the sample generated by the propagating elastic waves were monitored with an optical coherence tomography (OCT) microscope. Wave speeds were determined as the ratio of the excitation frequency (2–6 kHz) and the wavelength of the elastic wave measured with OCT. Wave speed measurements across frequencies were then used to construct phase velocity dispersion curves for beads produced at each combination of crosslinking times and pH (n = 6–10). For each frequency, the average wave speed at each excitation frequency for each crosslinking condition was used to estimate the sample frequency-dependent Young’s modulus with a finite-element-based steady state elastodynamic wave model^55^. Because wave speeds were independent of frequency, the Young’s moduli (E) estimates across the range of excitation frequencies were used to estimate the mean E and standard deviations. Further details of the OCE measurement technique and the finite element model are provided in the supplemental information (Supplementary Information, S.1.1).

We also estimated the apparent optical attenuation coefficient for each sample category using the structural OCT data. The OCT data represents local light scattering intensity. This means spatial contrast in the data is indicative of local changes in the refractive index, which is in turn correlated with the local polymer density in the sample^53^. We tracked the variation in the average attenuation coefficient with cross-linking time and pH, assuming that the light intensity decreases from a peak value at sample surface to a minimum detectable limit at some distance below the surface. Specifically, the average pixel intensity in the OCT image was first recorded near the sample surface, and this measurement was repeated along a transect of 100 pixels (92.1 μm) below the sample surface. Along this transect, the average intensity decreased linearly with distance below the surface. The slope of the decaying intensity, which we call the optical decay coefficient, is compared for the different cross-linking times and pH values.

### Physicochemical properties of hydrogel beads

To assess diffusive properties of beads produced under different crosslinking conditions, a dextran blue diffusive assay was developed. First, 0.5 mL of beads (as measured by volume displacement) were allowed to equilibrate at least 24 h with a 1% Dextran Blue (approx. 2000 kDa, Sigma-Aldrich, USA) solution. At the start of the diffusion assay, 0.5 mL of beads were put in 5 mL of synthetic medium in clear glass Balch tubes and well-mixed. Optical density at 600 nm (OD_600_) was measured in a spectrophotometer right after mixing. Subsequently Balch tubes were shaken at 120 rpm using a nearly horizontal angle, and OD_600_ was measured every 2 min for the first hour, every 10 min for the following 30 min, and every 20 min for the last hour. Measured values were corrected with the initial OD_600_ value. Assays were executed in triplicate for each hydrogel formulation.

### Hydrogel incubation

Hydrogel beads were incubated in shake flasks with regular liquid replacement to simulate beads in a bioreactor. Before the start of the experiment, hydrogel beads were washed with DI water. In 250 mL shake flasks, 25 mL of hydrogel beads—measured by volume displacement in a graduated cylinder—and 75 mL of medium was added. Shake flasks were incubated on a shaker at 200 rpm in a temperature-controlled room at 30 °C. Every 2–3 days, 15 mL of liquid was sampled for analysis, beads were strained, and 75 mL fresh medium was added.

Loss of fluorescent microspheres was quantified at each liquid exchange step using a Guava EasyCyte flow cytometer (Luminex, Austin, USA) equipped with a 50 mW 488 nm laser. Beads were quantified by using the green fluorescence signal (525 nm band pass filter). Loss of PVA from the hydrogel beads during incubation was quantified using a previously described blue iodine assay, modified to microwell plate^56^. In brief, each well was filled with 36.4 µL of sample, 163.3 µL of 4% boric acid solution, and 27.3 µL of iodine solution. The iodine solution was prepared by dissolving 250 mg KI and 127 mg I_2_ in 10 mL of MilliQ water and letting the solution dissolve overnight on a shaker at 30 °C. After addition of the iodine solution, the well plate was left to incubate for 20 min at room temperature before measurement on a CLARIOstar Plus microwell plate reader (BMG Labtech, Ortenberg, Germany). Each microwell plate was internally calibrated with a PVA standard series ranging from 0 to 100 mg PVA L^−1^. Loss of total polymer in the supernatant was measured using COD test kits following Standard Method 5220D with a lower detection limit of 3 mg COD L^−1^.

## Supplementary Information

Supplementary Information.

## Data Availability

The dataset supporting the conclusions of this article are available upon reasonable request from the corresponding author (P.C.).
