# Supplementary material for: Tailoring polyvinyl alcohol-sodium alginate (PVA-SA) hydrogel beads by controlling crosslinking pH and time
Source: Sci Rep. 2022 Dec 2;12:20822. doi: 10.1038/s41598-022-25111-7 (PMC9718846; doi:10.1038/s41598-022-25111-7)
Supplement: Supplementary file 1 — Supplementary Information. [file 41598_2022_25111_MOESM1_ESM.pdf]

SUPPLEMENTARY INFORMATION

Supplementary information to: Tailoring polyvinyl alcohol-sodium alginate (PVA-SA) hydrogel beads by controlling crosslinking pH and time

Pieter Candry[1,#,], Bruce J. Godfrey[1,#], Ziwei Wang[2], Fabrizio Sabba[3], Evan Dieppa[4], Julia Fudge[1], Oluwaseyi Balogun[2,5], George Wells[5], Mari-Karoliina Henriikka Winkler[1]*

[1] Civil and Environmental Engineering, University of Washington, 201 More Hall, Box 352700, Seattle, WA 98195-2700, USA

[2] Mechanical Engineering Department, Northwestern University, Evanston, IL 60208, United States

[3] Black & Veatch, KS, United States

[4] Theoretical and Applied Mechanics Program, Northwestern University, Evanston, IL 60208, United States

[5] Civil and Environmental Engineering Department, Northwestern University, Evanston, IL 60208, United States

[#] These authors contributed equally

* Correspondence to: Pieter Candry, pcandry@uw.edu, University of Washington, 201 More Hall, Box 352700, Seattle, WA 98195-2700, USA

S.1. Material & Methods

S.1.1. Optical coherence elastography (OCE)

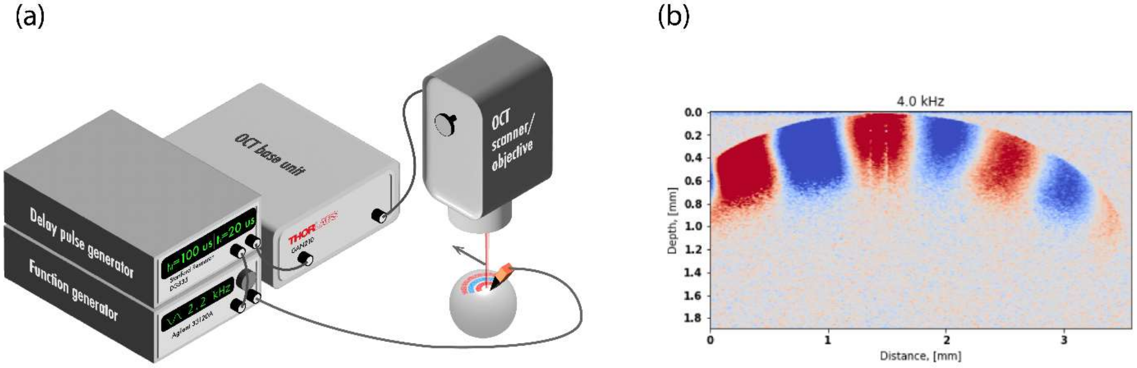

Figure S.1. (a) OCE experiment setup. The PVA-SA bead sample was placed under the OCT objective and was loaded with the piezoelectric transducer. (b) An example of OCE image captured from a PVA-SA bead at the excitation frequency of 4 kHz. Blue and red stripes show the local vertical displacement field going upwards and downwards.

Shown in Figure S.1(a), the optical coherence elastography (OCE) experiment setup consists of a commercial spectral-domain phase-sensitive optical coherence tomography (OCT) system (GAN210C1, Thorlabs), and a piezoelectric transducer stack (PK4JQP2, Thorlabs) driven by a function generator (33120A, Agilent). The default A-scan rate of the system is 36 kHz with 101 dB sensitivity. The laser source of the system has a center wavelength of 930 nm with 6 μm axial resolution and 2.9 mm imaging depth in air. A 3D printed polymeric tip triangular prism was glued to the transducer, and put in contact with the bead sample to provide oscillatory force load for harmonic elastic wave generation. The piezoelectric transducer and the OCT system were synchronized with a delay pulse generator (DG535, Stanford Research) to produce the steady-state image of the elastic wave field, for example, as shown in Figure S.1(b). The wave speed was then calculated from the OCE image via a spatial Fourier transform.

S.1.2. Finite-element-based circumferential wave dispersion model

Hydrogel beads in this study have spherical shapes, thus the elastic waves traveling along the surface are called circumferential waves[1]. In addition, the observed wavelengths from 2 kHz to 6 kHz are in the same order as the average radius of curvature of the samples, which means the curvature has considerable effects on the wave dispersion behaviors. Thus, a circumferential wave dispersion finite-element model was built in COMSOL Multiphysics to infer Young's modulus of the sample from the measured wave speed.

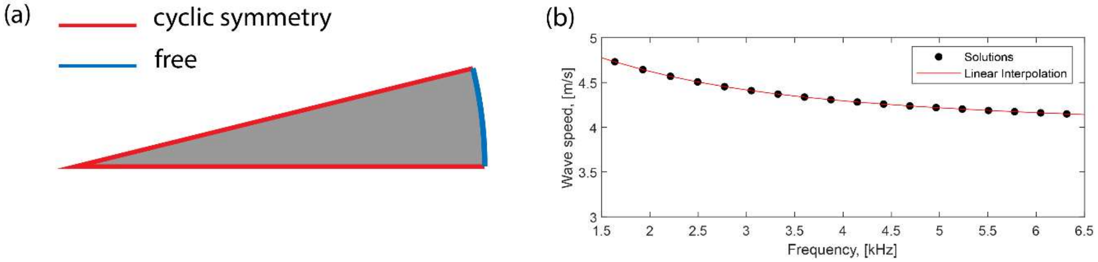

Figure S.2. (a) Finite element model (FEM) of circumferential dispersion; (b) An example of simulated wavespeed-frequency dispersion results. Black dots are eigenfrequency solutions from the FEM. The red line is the linear interpolation of the solution points.

As shown in Figure S.2(a), the bead sample was modeled as a circular sector with a free arc boundary, and two radii subjected to cyclic symmetry with a variable azimuthal mode number k. The circular angle was 1 degree with a radius r = 2.3 mm. The material of the bead model was assumed to be linear elastic with Poisson's ratio ν = 0.499, mass density ρ = 1024 kg/m^3, and Young's modulus E as a free parameter to be determined. By optimizing E and fitting the simulated wave speed ($c_s = 2\pi f r / k$) at frequency f to the OCE measured wave speed (c_e) at f, Young's modulus E of the sample at f can be determined. Note that since the f-c relation is implicit, k was swept from 1 to 30 for the eigenfrequency analysis to obtain a wide range of solutions of $c(f(k), k)$, and c_s at f was found via the interpolation. Figure S.2(b) shows an example of calculated wave speed points (black dots) and the linearly

interpolated wave speed curve (red line).

S.2. Results

S.2.1. Physical properties

Table S.1. Statistical comparison of particle size distributions (PSD) between different hydrogel production conditions. The non-parametric Wilcox test was used for group-by-group comparisons, and Bonferroni-adjusted p-values are reported: *, p<0.05; **, p<0.01; ***, p<0.001

Condition		pH 3			pH 4			pH 5		
		1 hour	2 hours	8 hours	1 hour	2 hours	8 hours	1 hour	2 hours	8 hours
pH 3	1 hour									
	2 hours									
	8 hours									
pH 4	1 hour									
	2 hours									
	8 hours									
pH 5	1 hour	**	***	***	***	***	***			
	2 hours	*	***	***	***	***	***			
	8 hours		***	***	***	***	***			

Table S.2. Statistical comparison of cavity area fractions between different hydrogel production conditions. The non-parametric Wilcox test was used for group-by-group comparisons, and Bonferroni-adjusted p-values are reported: *, p<0.05; **, p<0.01; ***, p<0.001s

Condition		pH 3			pH 4			pH 5		
		1 hour	2 hours	8 hours	1 hour	2 hours	8 hours	1 hour	2 hours	8 hours
pH 3	1 hour									
	2 hours									
	8 hours									
pH 4	1 hour									
	2 hours									
	8 hours									
pH 5	1 hour	**	*	**	***	***	**			
	2 hours	*		*	**	**	*			
	8 hours				*	*				

Table S.3. Statistical comparison of optical decay coefficients between different hydrogel production conditions. A pairwise t-test was used for group-by-group comparisons, and Bonferroni-adjusted p-values are reported: *, $p<0.05$; **, $p<0.01$; ***, $p<0.001$

Condition		pH 3			pH 4			pH 5		
		1 hour	2 hours	8 hours	1 hour	2 hours	8 hours	1 hour	2 hours	8 hours
pH 3	1 hour									
	2 hours									
	8 hours	**	**							
pH 4	1 hour			**						
	2 hours									
	8 hours									
pH 5	1 hour	***	***	***	***	***	***			
	2 hours	***	***	***	***	***	***			
	8 hours	***	***	***	***	***	***			

Table S.4. Statistical comparison of Young's modulus between different hydrogel production conditions. A pairwise t-test was used for group-by-group comparisons, and Bonferroni-adjusted p-values are reported: *, $p<0.05$; **, $p<0.01$; ***, $p<0.001$

Condition		pH 3			pH 4			pH 5		
		1 hour	2 hours	8 hours	1 hour	2 hours	8 hours	1 hour	2 hours	8 hours
pH 3	1 hour									
	2 hours	***								
	8 hours	***	**							
pH 4	1 hour	***	**							
	2 hours	***								
	8 hours	***	***							
pH 5	1 hour	*	***	***	***	***	***			
	2 hours		***	***	***	***	***			
	8 hours		***	***	***	***	***			

Table S.5. Statistical comparison of final OD600 from Dextran Blue diffusion assays between different hydrogel production conditions. A pairwise t-test was used for group-by-group comparisons, and Bonferroni-adjusted p-values are reported: *, p<0.05; **, p<0.01; ***, p<0.001

Condition		pH 3			pH 4			pH 5		
		1 hour	2 hours	8 hours	1 hour	2 hours	8 hours	1 hour	2 hours	8 hours
pH 3	1 hour									
	2 hours	*								
	8 hours	*								
pH 4	1 hour	***								
	2 hours	***								
	8 hours	***	**	**						
pH 5	1 hour	***	***	***		*				
	2 hours	***	***	***		*				
	8 hours	***	**	**						

Table S.6. Statistical comparison of diffusion rates from Dextran Blue diffusion assays between different hydrogel production conditions. A pairwise t-test was used for group-by-group comparisons, and Bonferroni-adjusted p-values are reported: *, $p<0.05$; **, $p<0.01$; ***, $p<0.001$

Condition		pH 3			pH 4			pH 5		
		1 hour	2 hours	8 hours	1 hour	2 hours	8 hours	1 hour	2 hours	8 hours
pH 3	1 hour									
	2 hours									
	8 hours	**								
pH 4	1 hour	***	**	*						
	2 hours	***								
	8 hours	***	***	**						
pH 5	1 hour	***	***	***						
	2 hours	***	***	***		**				
	8 hours	***	***	**						

Table S.7. Statistical comparison of microsphere loss across a 28-day incubation with regular media replacement. A pairwise t-test was used for group-by-group comparisons, and Bonferroni-adjusted p-values are reported: *, $p<0.05$; **, $p<0.01$; ***, $p<0.001$

Condition		pH 3			pH 4			pH 5		
		1 hour	2 hours	8 hours	1 hour	2 hours	8 hours	1 hour	2 hours	8 hours
pH 3	1 hour									
	2 hours									
	8 hours									
pH 4	1 hour	**								
	2 hours	*								
	8 hours	**								
pH 5	1 hour		*	**	***	***	***			
	2 hours		*	**	***	***	***			
	8 hours		*	**	***	***	***			

Table S.8. Statistical comparison of PVA-loss across a 28-day incubation with regular media replacement. A pairwise t-test was used for group-by-group comparisons, and Bonferroni-adjusted p-values are reported: *, $p<0.05$; **, $p<0.01$; ***, $p<0.001$

Condition		pH 3			pH 4			pH 5		
		1 hour	2 hours	8 hours	1 hour	2 hours	8 hours	1 hour	2 hours	8 hours
pH 3	1 hour									
	2 hours									
	8 hours									
pH 4	1 hour	***	***	***						
	2 hours	***	**	***						
	8 hours	***	***	***						
pH 5	1 hour	***	***	***		**	*			
	2 hours	***	***	***	***	***	***	**		
	8 hours	***	***	***	***	***	***	***	***	

Table S.9. Statistical comparison of total polymer loss (expressed as COD) across a 28-day incubation with regular media replacement. A pairwise t-test was used for group-by-group comparisons, and Bonferroni-adjusted p-values are reported: *, $p<0.05$; **, $p<0.01$; ***, $p<0.001$

Condition		pH 3			pH 4			pH 5		
		1 hour	2 hours	8 hours	1 hour	2 hours	8 hours	1 hour	2 hours	8 hours
pH 3	1 hour									
	2 hours	***								
	8 hours	***								
pH 4	1 hour	***	***	**						
	2 hours	***	**							
	8 hours	***	***	***						
pH 5	1 hour	***	***	***						
	2 hours	***	***	***	**	***	**			
	8 hours	***	***	***	***	***	***	***	**	

S.3. References

(1) Qu, J.; Berthelot, Y.; Li, Z. Dispersion of Guided Circumferential Waves in a Circular Annulus. *Review of Progress in Quantitative Nondestructive Evaluation* **1996**, 169–176. https://doi.org/10.1007/978-1-4613-0383-1_21.
